# Efficacy and Safety of Immune Checkpoint Inhibitors for Lung Cancer Complicated by Interstitial Pneumonia: The CS‐Lung 003 Study, Branch Number 28

**DOI:** 10.1111/1759-7714.70399

**Published:** 2026-10-15

**Authors:** Kosuke Hamai, Nobuhiro Kanaji, Naoki Watanabe, Nobuhisa Ishikawa, Sayaka Ueno, Takeshi Masuda, Noboru Hattori, Masahiro Yamasaki, Naoko Matsumoto, Masamoto Nakanishi, Nobukazu Fujimoto, Nagio Takigawa, Kazuya Nishii, Katsuyuki Hotta, Katsuyuki Kiura

**Affiliations:** ^1^ Department of Respiratory Medicine JA Onomichi General Hospital Hiroshima Japan; ^2^ Department of Respiratory Medicine Hiroshima Prefectural Hospital Hiroshima Japan; ^3^ Department of Internal Medicine, Division of Hematology, Rheumatology and Respiratory Medicine, Faculty of Medicine Kagawa University Kida‐gun Kagawa Japan; ^4^ Department of Respiratory Medicine Hiroshima University Hospital Hiroshima Japan; ^5^ Department of Respiratory Medicine Hiroshima Red Cross Hospital and Atomic‐Bomb Survivors Hospital Hiroshima Japan; ^6^ Department of Respiratory Medicine Okayama University Hospital Okayama Japan; ^7^ Department of Medical Oncology and Medicine Okayama Rosai Hospital Okayama Japan; ^8^ Department of General Internal Medicine 4 Kawasaki Medical School Okayama Japan; ^9^ Department of Respiratory Medicine National Hospital Organization Iwakuni Clinical Center Yamaguchi Japan; ^10^ Center for Innovative Clinical Medicine Okayama University Okayama Japan

**Keywords:** drug‐induced pneumonitis, immune checkpoint inhibitors, interstitial pneumonia, lung cancer

## Abstract

**Background:**

Immune checkpoint inhibitors (ICIs) improve the prognosis of patients with previously treated non‐small cell lung cancer (NSCLC). However, the efficacy and safety of ICIs for patients with NSCLC complicated with interstitial pneumonia (IP) remain unknown. This study aimed to analyze patients with NSCLC and IP treated with ICIs and to investigate the efficacy and exacerbation frequency of ICI therapy.

**Methods:**

We prospectively enrolled patients with NSCLC and IP at multiple centers and examined progression‐free survival (PFS) and overall survival (OS) using the Kaplan–Meier method.

**Results:**

The median PFS was 5.8 months, and the median OS was 9.7 months. ICI‐related lung injury was observed in seven of the 19 patients (36.8%). Pneumonitis was observed in one of five patients with a usual interstitial pneumonia (UIP) pattern and in six of 14 patients with a non‐UIP pattern; however, there was no significant difference in onset frequency. Furthermore, we observed no significant difference in PFS between patients with and without ICI‐related pneumonitis (6.8 vs. 3.3 months, respectively; *p* = 0.826). OS tended to be shorter in patients with ICI‐related pneumonitis, but the difference was not significant (8.0 vs. 16.7 months, respectively; *p* = 0.172).

**Conclusions:**

The use of ICIs in patients with NSCLC and IP produces long‐term responses in some cases; however, there is a high possibility of ICI‐related pneumonitis. In cases where the condition worsens, post‐treatment may be difficult, and the prognosis may be poor.

## Graphical abstract

In the CS‐Lung003 trial, branch 28, which retrospectively investigated the efficacy and safety of ICIs in previously treated NSCLC complicated with IP, the median PFS was 5.8 months, and the median OS was 9.7 months. No significant difference was observed in PFS between patients with and without ICI‐related pneumonitis (6.8 vs. 3.3 months, respectively; *p* = 0.826). OS tended to be shorter in patients with ICI‐related pneumonitis, but the difference was not significant (8.0 vs. 16.7 months, respectively; *p* = 0.172).

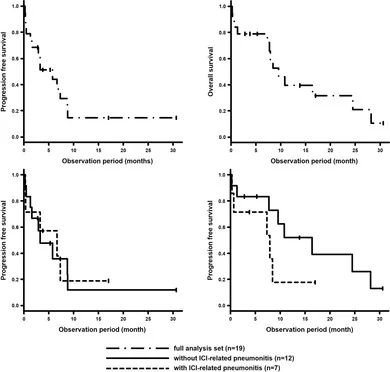

## 1 Introduction

Single‐agent immune checkpoint inhibitors (ICIs) have been demonstrated to significantly extend the overall survival (OS) of patients with recurrent non‐small cell lung cancer (NSCLC) compared to docetaxel alone (approximately 12 months vs. 9 months in ICIs and docetaxel, respectively) [1, 2, 3]. However, ICIs can cause immune‐related adverse events, including drug‐induced pneumonitis, that can sometimes be fatal. The incidence of drug‐induced pneumonitis is reportedly higher in patients with pre‐existing pulmonary fibrosis [4].

Idiopathic pulmonary fibrosis (IPF) is an idiopathic interstitial pneumonia (IP) with a poor prognosis, and acute exacerbations (AEs) account for 40% of deaths [5]. Furthermore, approximately 40% of patients with IP associated with progressive fibrosis other than IPF die from AEs [6]. Drug‐induced lung injury often occurs when ICIs are used to treat lung cancer in patients with IP. The Japanese Guideline for the Treatment of Idiopathic Pulmonary Fibrosis 2023 states that administering ICIs to patients with lung cancer complicated by IP is not recommended but may be effective for some patients. Zhang et al. reported that the objective response rate to ICIs was higher in patients with lung cancer with existing interstitial opacities than in those without (odds ratio 1.99, 95% confidence interval [CI] 1.31–3.00). However, the incidence of drug‐induced pneumonitis was higher (27% vs. 10%, respectively) [7]. Therefore, the efficacy and safety of ICIs in patients with lung cancer and IP remain unknown.

This multicenter prospective observational study aimed to analyze patients with NSCLC and IP treated with ICIs and to investigate the efficacy of ICIs and the frequency of ICI‐related pneumonitis.

## 2 Methods

### 2.1 Data Source

In the CS‐Lung003 trial, an umbrella‐type prospective lung cancer patient registry, we prospectively enrolled all patients with lung cancer who had provided written informed consent from March 2017 at multiple centers (UMIN000026696) [8]. We then selected those diagnosed with IP by attending physicians who received ICI monotherapy after second‐line treatment for NSCLC between March 2017 and August 2020 (Figure 1). The background classification of IP was based on ATS/ERS/JRS/ALAT 2018 [9]. The usual interstitial pneumonia (UIP) pattern exhibits subpleural and basal‐predominant shadows that are often heterogeneous. It is also characterized by honeycombing, traction bronchiectasis/bronchiolectasis, and irregular thickening of the alveolar septa. Attending pulmonary physicians distinguished between UIP and non‐UIP patterns at the time of registration. There were no restrictions placed on registration based on pulmonary function tests. AE of IP was defined as an acute, clinically significant respiratory deterioration (e.g., changes in oxygen saturation) of unidentifiable cause [10]. Because all patients in this study received ICIs, the attending physician distinguished ICI‐related pneumonitis from AE of pre‐existing IP and ICI‐associated drug‐induced pneumonitis. AE of IP was defined as worsening of pre‐existing interstitial opacities, whereas drug‐induced pneumonitis was defined by the appearance of opacities with new morphological features or new opacities in other regions. Adverse events of chemotherapy were based on the Common Terminology Criteria for Adverse Events ver. 5.0.

**FIGURE 1:**
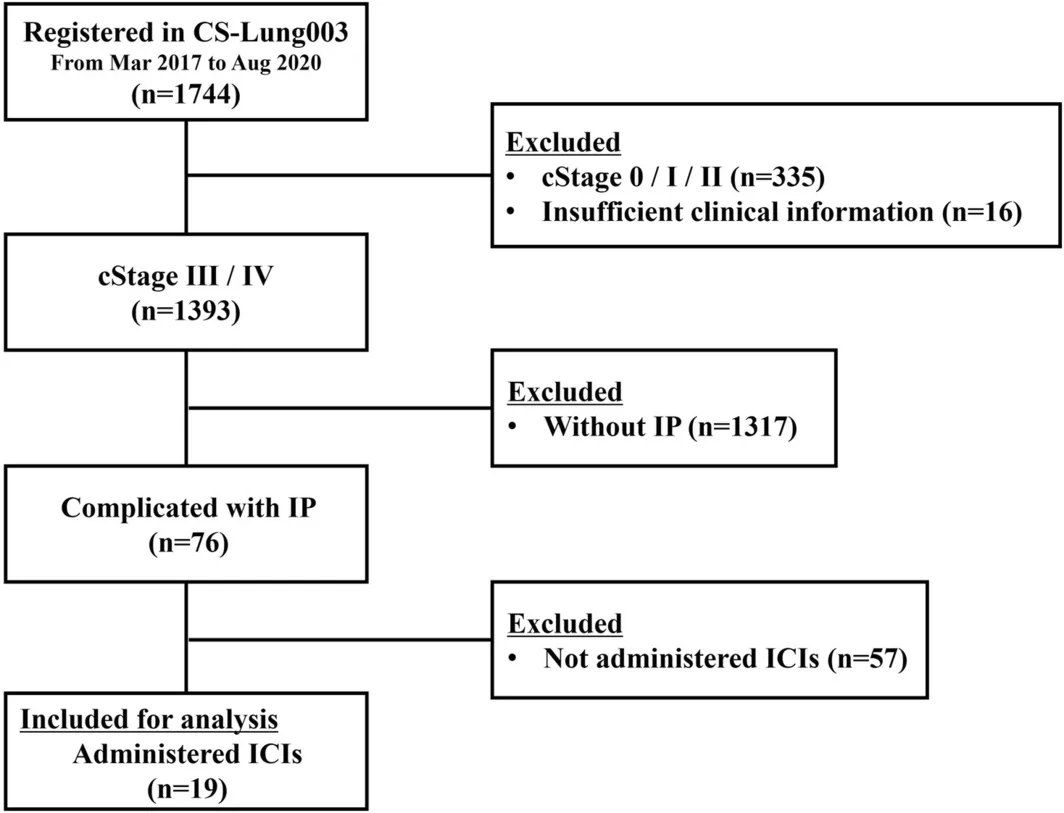
Consort diagram showing screening and exclusions from the CS‐Lung003 registry.

The study protocol was approved by the Ethics Committee of Okayama University Hospital (approval No. 1703‐055, approval date 17/Mar/2017) and the institutional review board of each participating hospital. Written informed consent was obtained from all participants. The study was conducted in accordance with the Declaration of Helsinki and the relevant Japanese regulations.

### 2.2 Statistical Analysis

Disease progression was assessed at the discretion of the attending physician. The interval for assessing efficacy was not specified, as this trial was an observational study. Therefore, the interval between computed tomography (CT) scans to assess efficacy varied depending on the case. Progression‐free survival (PFS) was defined as the time from the start of ICI treatment to disease progression, and OS was defined as the time from the start of ICI treatment to death. Time to onset of pneumonitis was defined as the time from ICI initiation to onset of drug‐associated pneumonitis. Kaplan–Meier curves were used to determine PFS, OS, and time to pneumonitis onset. All statistical analyses were performed using R software (R Foundation for Statistical Computing, Vienna, Austria).

## 3 Results

### 3.1 Patient Characteristics

Nineteen patients with lung cancer complicated with IP were prospectively enrolled from nine institutions. The median patient age was 71 years, and 18 patients were men. Eight patients had adenocarcinoma, nine had squamous cell carcinoma, and two had NSCLC, not otherwise specified. All patients possessed a history of smoking, and eight were current smokers. The administered ICIs were nivolumab in seven cases, pembrolizumab in eight cases, and atezolizumab in four cases.

Regarding existing IP, five cases exhibited a UIP pattern, and 14 exhibited a non‐UIP pattern. The mean Krebs von den Lungen‐6 (KL‐6) value was 577 IU/L. Only 16 patients underwent pulmonary function tests. The mean forced vital capacity (FVC) was 86.0%. The diffusion capacity of lung carbon monoxide (DL_CO_) was measured in 10 patients, with a mean of 69.9%. Only two patients received nintedanib at ICI initiation, and no patients had received corticosteroids or immunosuppressants prior to starting ICIs. One patient presented with IP associated with systemic sclerosis, whereas two patients tested positive for autoantibodies, although the specific connective tissue disease remained unclear (Table 1).

**TABLE 1:** Patient characteristics (*n* = 19).

Age, median (range)	71 (63–85)
Sex	
Male	18
Female	1
Histology	
Adeno	8
Squamous	9
Other	2
Stage	
III	10
IV	9
ICIs	
Nivolumab	7
Pembrolizumab	8
Atezolizumab	4
Treatment line	
2nd	6
3rd	8
4th	5
Nintedanib	
Yes	2
No	17
Corticosteroids or immunosuppressants	
Yes	0
No	19
Collagen disease or autoimmune antibody	
Yes	3
No	14
Unknown	2
PD‐L1	
< 1%	2
1%–49%	6
≥ 50%	7
Unknown	4
Smoking	
Current	8
Ex	11
KL‐6, IU/L (median)	183 → 10 000 (443)
%VC, % (mean ± SD)	82.7 ± 14.6
FEV1.0, L (mean ± SD)	2.2 ± 0.5
FEV1/FVC, % (mean ± SD)	76.6 ± 8.3
%DLco, % (mean ± SD)	69.9 ± 31.2

Abbreviations: DLco: diffusing capacity of the lung carbon monoxide, FEV1.0: forced expiratory volume 1.0, FVC: forced vital capacity, ICS: immune checkpoint inhibitor, KL‐6: Klebs von den Lungen‐6, PD‐L1: program death‐ligand 1, VC: vital capacity.

### 3.2 Efficacy

The median PFS was 5.8 months (95% CI 1.3–8.9) (Figure 2A), and the median OS was 9.7 months (95% CI 7.4–24.9) (Figure 2B). Of the 19 patients, two were able to continue ICIs for more than 1 year, and approximately 30% survived for more than 2 years after starting ICI treatment.

**FIGURE 2:**
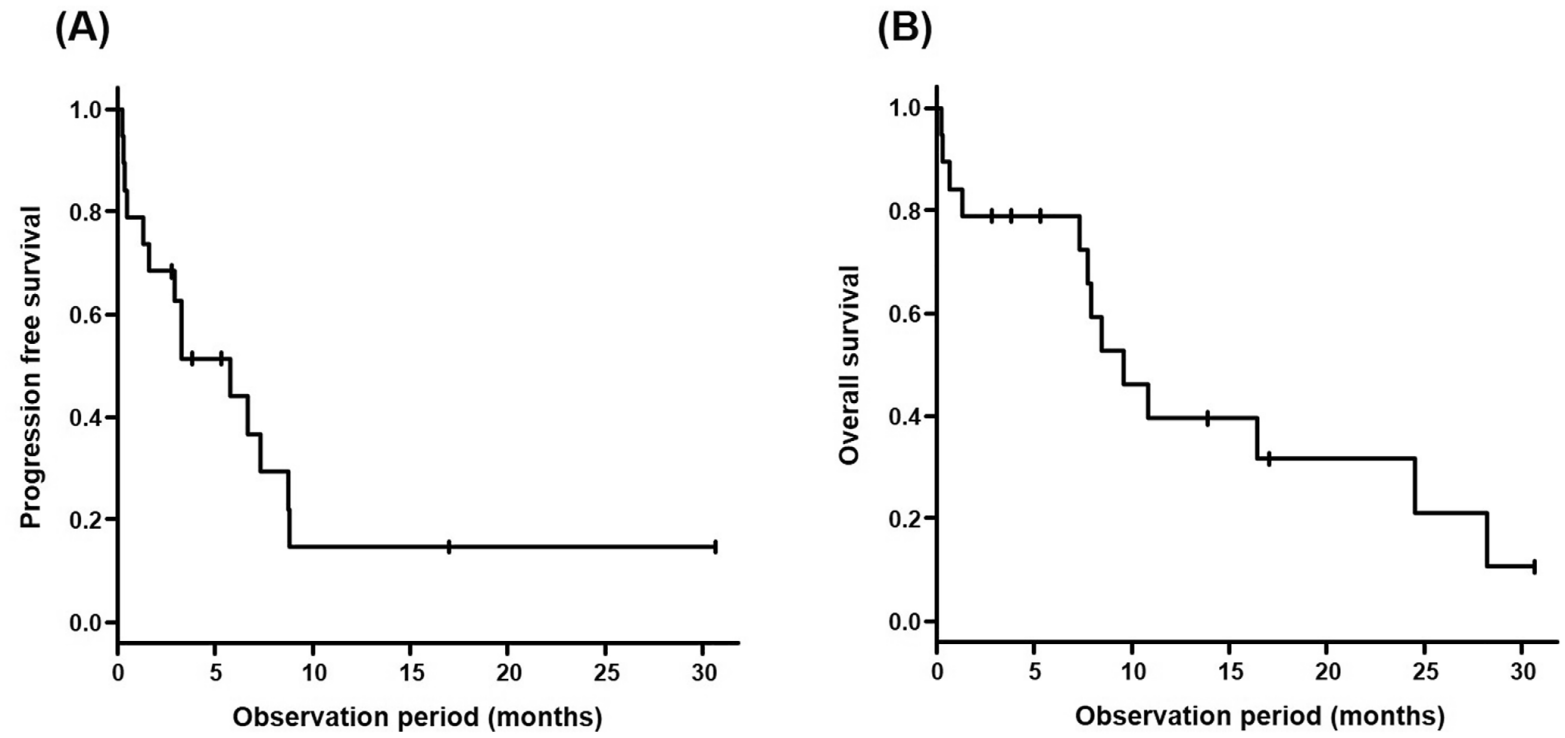
Kaplan–Meier curves indicating progression‐free survival (A) and overall survival (B) in all enrolled patients.

Patients with ICI‐related pneumonitis experienced a higher PFS (6.8 vs. 3.3 months, respectively, *p* = 0.826) (Figure 3A) and lower OS (8.0 vs. 16.7 months, respectively, *p* = 0.172) (Figure 3B) than those without ICI‐related pneumonitis, but the difference was not significant. Only one of seven patients who experienced ICI‐related pneumonitis received subsequent chemotherapy, compared to four of 12 patients who did not experience ICI‐related pneumonitis.

**FIGURE 3:**
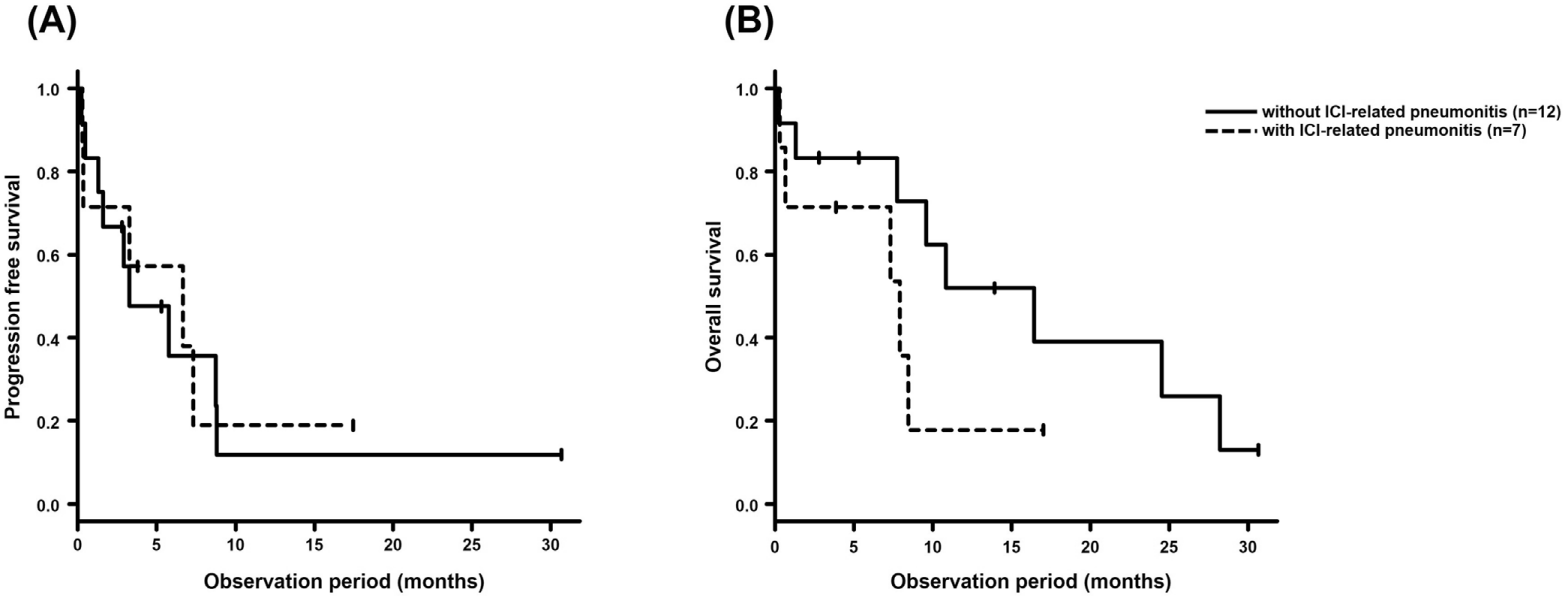
Kaplan–Meier curves indicating progression‐free survival (A) and overall survival (B) between patients with and without ICI‐related pneumonitis.

### 3.3 Adverse Events

ICI‐related pneumonitis was observed in seven of 19 patients (36.8%) (Table 2). Among these cases, the attending physician classified four as drug‐induced pneumonitis, two as AE of pre‐existing IP, and one as unclassifiable. Regarding the pneumonitis patterns, one case of drug‐induced pneumonitis showed an organizing pneumonia pattern, and one case of AE of IP showed a non‐specific interstitial pneumonia pattern; the remaining cases exhibited a diffuse alveolar damage (DAD) pattern. One patient with the DAD pattern had Grade 5 pneumonitis, whereas the remaining six patients required treatment postponement, dose reduction, or discontinuation. Drug‐induced pneumonitis with a DAD pattern occurred in one of the two patients treated with nintedanib. Pneumonitis occurred in one of five patients with a baseline UIP pattern and in six of 14 patients with a non‐UIP pattern; however, the difference in incidence was not significant (Figure 4). Additionally, hypopituitarism of Grade 3 requiring hormone replacement, myositis of Grade 3 requiring oral corticosteroids, and adrenal insufficiency of Grade 1 were observed in one case each.

**TABLE 2:** Frequency of immune related adverse events for immune checkpoint inhibitor (ICI).

Pneumonitis	36.8%
Adrenal insufficiency	5.3%
Hypopituitarism	5.3%
Myositis	5.3%
Others	5.3%

**FIGURE 4:**
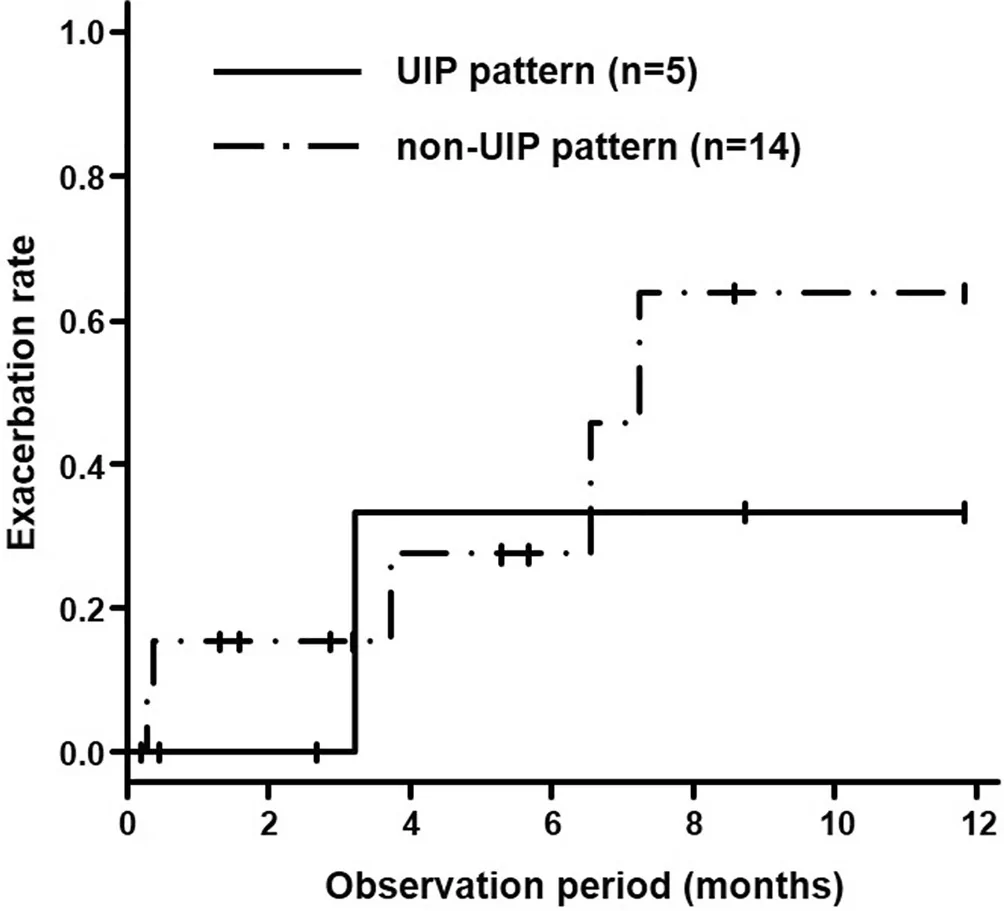
Kaplan–Meier curves indicating the onset of exacerbation of existing interstitial pneumonia (IP) between patients with usual interstitial pneumonia (UIP) pattern pneumonitis and those with non‐UIP pattern. The median duration of ICI‐related pneumonitis onset in those with non‐UIP pattern was 7.3 months, but that in those with UIP pattern was not reached.

## 4 Discussion

Although ICI therapy for the treatment of lung cancer with IP can improve the prognosis in some cases, it can cause drug‐related pneumonitis. Ikeda et al. conducted a Phase II study to verify the efficacy and safety of atezolizumab in patients with previously treated lung cancer associated with IP, reporting that IP worsened in five of 17 patients [11]. In contrast, Fujimoto et al. conducted a Phase II study to verify the efficacy and safety of nivolumab for previously treated lung cancer complicated by IP. They observed no exacerbation of IP and a 6‐month PFS rate of 56% [12]. The enrollment criteria for Fujimoto et al.'s study were a possible UIP pattern or inconsistent UIP pattern, absence of honeycombing, negative results for autoantibodies suggestive of collagen disease, and an FVC > 80%. Based on the results of comparing these two tests, the authors proposed the absence of honeycombing, negative for autoantibodies suggestive of collagen disease, and FVC > 80% as the “HAV criteria.” This condition was proposed to ensure the safe administration of ICIs for lung cancer.

Ikeda and Fujimoto classified IP based on the presence or absence of honeycombing. However, some studies have demonstrated that ground‐glass opacities (GGOs) are more important than honeycombing when administering ICIs to patients with lung cancer and IP. In a retrospective study of lung cancer with IP treated with ICIs, Nishiyama et al. reported that the incidence of pneumonitis was 15% and that GGOs were a significant risk factor [13]. Daido et al. also reported that interstitial lung abnormalities were an independent risk factor for pneumonitis when using durvalumab after chemoradiotherapy, and the presence of GGOs was a significant factor [14]. In our cohort, five of the 19 patients exhibited a definite UIP pattern with honeycombing, and three had concomitant collagen vascular disease or tested positive for autoantibodies. Although the mean %FVC was 86.2%, eight patients had an FVC of less than 80%. The study enrolled patients deemed by physicians to be at relatively low risk for ICI treatment, but the cohort did not meet the HAV criteria for a low‐risk population. Consistent with this, ICI‐related pneumonitis occurred in seven of the 19 cases (36.8%). However, the frequency of exacerbation did not differ between patients with and without a definite UIP pattern featuring or not featuring honeycombing. Although many non‐UIP pattern cases may have exhibited GGOs, detailed imaging characteristics were not evaluated in this study.

A recent multicenter retrospective observational study reported drug‐induced pneumonitis in 61 of 200 patients (30.5%) with lung cancer and IP who received ICIs, including combination therapy with cytotoxic chemotherapy. Additionally, patients with Grade 1–2 pneumonitis tended to have better treatment response to ICIs than those with Grade 3 or higher pneumonitis or those without pneumonitis [15]. This study indicated no significant difference between cases in which ICIs were discontinued owing to worsening IP and those in which they were not discontinued due to the small number of cases. Additionally, the prognosis in cases with ICI‐related pneumonitis tended to be poorer. The antitumor effects of ICIs may last for a long time in cases where treatment is discontinued owing to immune‐related adverse events such as lung damage [16, 17]. However, in our study, only one of seven patients who experienced ICI‐related pneumonitis received post‐treatment, compared to 4 of 12 patients who did not experience pneumonitis. Among the seven cases of ICI‐related pneumonitis, one was Grade 5, and the remaining six required either postponement, dose reduction, or discontinuation of subsequent therapy. The inability to administer subsequent therapy due to the onset of pneumonitis may be associated with a poorer prognosis.

This study possessed some limitations. First, the sample size was small, as this was a branch study of a multicenter prospective observational trial of patients with lung cancer. In addition, the CT scans intervals were not standardized, limiting interpretation of PFS and OS. Second, characterization of underlying IP was limited because this registry study was designed for advanced lung cancer rather than detailed IP evaluation. UIP patterns and non‐UIP patterns were classified based on the presence or absence of honeycombing, whereas other imaging features, such as GGOs, were not systematically assessed. Furthermore, the tissue evaluation including surgical biopsy or blood‐alveolar lavage was not performed as described in IP guidelines, preventing detailed classification of IP. We also did not evaluate the presence or absence of antigen exposure; thus, hypersensitivity pneumonitis cannot be ruled out. Pulmonary function tests were only performed in some cases, making it difficult to accurately determine the severity of the IP. Although almost all patients presumably underwent positron emission tomography‐CT for staging purposes, ^18^F‐fluorodeoxyglucose uptake in pre‐existing IP area was not evaluated, limiting assessment of disease severity. Only cases where physicians determined that ICI administration was relatively safe were included; even in such cases, the rate of ICI‐related pneumonitis is high at 36.8%, suggesting that unmeasurable differences in the underlying pulmonary etiology and severity contributed to the high incidence of pneumonitis and subsequent treatment challenges. Third, only a small number of patients received concomitantly nintedanib, preventing evaluation of its potential effects. A previous Phase III trial did not indicate that adding nintedanib to carboplatin plus paclitaxel in patients with lung cancer with IP reduced drug‐induced pneumonia [18]. However, nintedanib prevented the decline in FVC in patients with IPF and significantly reduced the time to the first acute exacerbation [19]. Therefore, the concomitant use of nintedanib may reduce the incidence of drug‐induced pneumonitis. Only two cases in this study received nintedanib, one of whom developed ICI‐related pneumonitis. The efficacy of nintedanib cannot be evaluated in this small cohort.

When ICIs are administered to patients with lung cancer complicated by IP, the IP can worsen, post‐treatment can be difficult, and the prognosis can deteriorate. Therefore, the indication for treatment should be carefully considered. Larger prospective randomized studies are warranted to clarify the efficacy and safety of ICI treatment in patients with lung cancer complicated by IP.

## 5 Conclusions

The use of ICI therapy in patients with lung cancer and IP produced long‐term responses in some cases; however, there is a high possibility of ICI‐related pneumonitis. Therefore, post‐treatment ICI therapy may be difficult in cases where the condition worsens, and the prognosis may be poor.

## Author Contributions

**Kosuke Hamai:** conceptualization, data curation, formal analysis, writing – original draft. **Nobuhiro Kanaji:** data curation, writing – review and editing. **Naoki Watanabe:** data curation, writing – review and editing. **Nobuhisa Ishikawa:** conceptualization, data curation, validation, writing – review and editing. **Sayaka Ueno:** data curation, writing – review and editing. **Takeshi Masuda:** writing – review and editing, data curation. **Noboru Hattori:** data curation, writing – review and editing. **Masahiro Yamasaki:** data curation, writing – review and editing. **Naoko Matsumoto:** data curation, writing – review and editing. **Masamoto Nakanishi:** data curation, writing – review and editing. **Nobukazu Fujimoto:** data curation, writing – review and editing. **Nagio Takigawa:** data curation, writing – review and editing. **Kazuya Nishii:** conceptualization, data curation, writing – review and editing. **Katsuyuki Hotta:** data curation, writing – review and editing. **Katsuyuki Kiura:** conceptualization, writing – review and editing.

## Funding

The authors have nothing to report.

## Disclosure

Generative AI was not used in the preparation of this manuscript.

## Conflicts of Interest

Kosuke Hamai has received honoraria from AstraZeneca (Japan). Nobuhisa Ishikawa received honoraria from AstraZeneca and Boehringer Ingelheim Co. Ltd. Noboru Hattori received honoraria from Chugai Pharmaceutical Co. Ltd., AstraZeneca, and Boehringer Ingelheim Co. Ltd. Nobukazu Fujimoto has received honoraria from AstraZeneca and GlaxoSmithKline. Nagio Takigawa received funding from Eli Lilly Co. Katsuyuki Hotta received research funding from MSD, Chugai Pharmaceutical Co. Ltd., AstraZeneca. and Bristol Myers Squibb Co. Ltd. The other authors declare no conflicts of interest.

## Data Availability

The data that support the findings of this study are available from the corresponding author upon reasonable request.
